# The role of *Atp2a2*-mediated calcium imbalance and endoplasmic reticulum stress in hydrocortisone-induced neurotoxicity

**DOI:** 10.1016/j.cstres.2025.100112

**Published:** 2025-09-17

**Authors:** Weihua Kong, Pei Jiang, Xinglu Miao, Ben Sang, Shunxin Hu, Lei Feng

**Affiliations:** 1 Institute of Central Nervous Vascular Injury and Repair, Jining Academy of Medical Sciences, Jining, China; 2 Jining Key Laboratory of Stroke and Neural Repair, Jining, China; 3 Translational Pharmaceutical Laboratory, Jining First People's Hospital, Jining, Shandong, China; 4 Department of Neurosurgery, Jining First People's Hospital, Jining, Shandong, China

**Keywords:** Zebrafish, Glucocorticoids, Neurotoxicity, Endoplasmic reticulum stress, Mitochondrial dysfunction, Calcium homeostasis

## Abstract

Glucocorticoids (GCs), as commonly used anti-inflammatory and immunosuppressive drugs, may induce neurotoxicity with long-term use, although the specific mechanisms remain unclear. This study utilized zebrafish as a model to investigate the mechanisms and potential intervention targets of hydrocortisone (HC)-induced neurotoxicity. Transcriptome analysis revealed that HC exposure significantly downregulated the expression of *Atp2a2* (encoding the endoplasmic reticulum calcium pump SERCA2). Functional experiments confirmed that HC disrupts cellular calcium homeostasis: endoplasmic reticulum Ca²⁺ levels decreased, mitochondrial Ca²⁺ accumulation occurred, accompanied by mitochondrial membrane potential depolarization, increased reactive oxygen species (ROS) generation, and cell apoptosis. Additionally, fluorescent signals in brain and spinal cord neurons were weakened, and significant decreases in movement distance, time, and average speed were observed. Intervention experiments with the GR antagonist RU486 and the SERCA2 activator demonstrated that both could partially restore calcium homeostasis, reduce ROS and apoptosis, and improve motor behavior. The findings revealed that HC disrupted calcium homeostasis by downregulating *Atp2a2*, activating endoplasmic reticulum stress, and triggering mitochondrial dysfunction, ultimately leading to neuronal damage and behavioral abnormalities. SERCA2 may serve as a potential target for alleviating GC-associated neurotoxicity, and this study provides experimental evidence for elucidating its mechanisms.

## Graphical abstract

**Figure:**
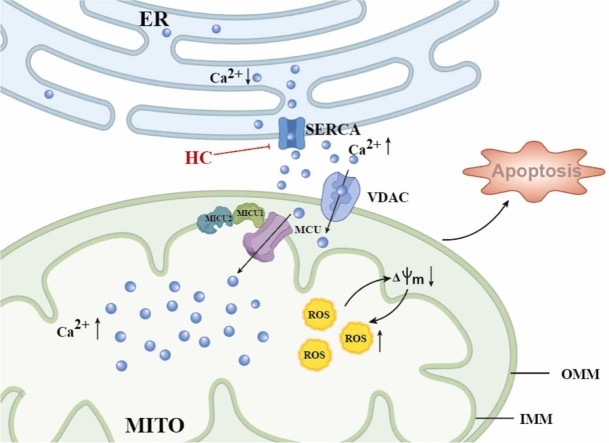

## Introduction

Glucocorticoids (GCs) are the most commonly used anti-inflammatory and immunosuppressive drugs in clinical practice, playing an irreplaceable role in the treatment of asthma, rheumatoid arthritis, and autoimmune diseases. However, long-term or high-dose use of GCs may induce significant neuropsychiatric adverse effects, including affective disorders such as depression and mania, as well as symptoms such as delirium and behavioral changes.^1^, ^2^, ^3^ A systematic review and meta-analysis of 49 studies revealed that the incidence of depression among GCs users was 22%, mania 11%, delirium 16%, and behavioral changes as high as 52%.^4^ Previous studies have reported that GC-induced neurotoxicity is closely associated with hippocampal neuronal apoptosis and impaired synaptic plasticity,^5^, ^6^ and that mice with glucocorticoid receptor (GR) gene mutations exhibit significant depressive and anxiety-like behaviors.^7^, ^8^, ^9^ Although the clinical hazards of GCs have been widely recognized, the specific molecular mechanisms underlying their neurotoxicity remain unclear, limiting the development of targeted neuroprotective strategies.

Calcium ions (Ca²⁺), as critical second messengers within cells, are involved in regulating essential physiological processes such as neuronal proliferation, synapse formation, and neurotransmitter release. Imbalance in calcium homeostasis is one of the core pathological mechanisms underlying neurotoxicity.^10^, ^11^ The endoplasmic reticulum (ER), which serves as the largest intracellular calcium reservoir, exhibits a significantly higher luminal Ca²⁺ concentration compared to the cytosol. This concentration gradient is sustained by calcium transport proteins on the ER membrane. Among these, *Atp2a2* gene encodes the sarcoplasmic/endoplasmic reticulum calcium ATPase 2 (SERCA2), a crucial calcium pump that actively transports Ca²⁺ from the cytosol into the ER lumen, thereby maintaining ER calcium homeostasis through ATP hydrolysis.^12^ Dysfunction of SERCA2 can directly lead to depletion of ER calcium, disrupting the protein folding microenvironment, and triggering the accumulation of unfolded proteins, thereby activating the unfolded protein response. During the initial adaptive phase, the unfolded protein response enhances protein folding capacity by upregulating molecular chaperones.^13^ If the stress persists and enters the terminal phase, proapoptotic pathways such as C/EBP homologous protein and caspases are activated, ultimately inducing cell death.^14^ Numerous studies have confirmed that defects in *Atp2a2*/SERCA2 function are closely associated with neurodegenerative diseases. In Alzheimer's disease, downregulation of SERCA2 expression accelerates tau protein phosphorylation and Aβ deposition;^15^, ^16^ in Parkinson's disease, reduced SERCA2 activity in substantia nigra neurons leads to dopaminergic neuronal apoptosis.^17^ Additionally, ischemic stroke-induced neuronal damage is accompanied by inhibition of SERCA2,^18^ indicating SERCA2 as a key target for intervention in neuronal damage.

Hydrocortisone (HC), an endogenous GC, exerts its biological effects primarily through genomic mechanisms mediated by the GR. Upon binding to HC, GR translocates to the nucleus and regulates the transcription of downstream target genes.^19^ Transcriptome sequencing analysis in this study revealed that the expression of Atp2a2, a gene associated with the endoplasmic reticulum stress (ERS) pathway, underwent significant alterations following HC exposure. It is known that Atp2a2 encodes SERCA2, a key protein regulating intracellular calcium homeostasis. Given the close relationship between ERS and calcium dysregulation, we preliminarily hypothesize that Atp2a2-mediated calcium transport dysfunction may be involved in the HC-induced neurotoxicity process. To validate this hypothesis, this study employed two tool molecules. Mifepristone (RU486), a synthetic steroid compound, competitively binds to the GR, thereby blocking the activation of the GC-mediated GR signaling pathway. In zebrafish models, it has been demonstrated to specifically inhibit GR-dependent transcription and enable real-time monitoring of GR signaling dynamics.^20^ Small-molecule SERCA2 activators relieve the inhibition of SERCA2 by competitively binding to phospholamban, thereby improving calcium cycling and diastolic function in cardiomyocytes.^21^ Based on its efficacy in myocardial repair, this study further evaluates its intervention effects on calcium homeostasis imbalance in the neurotoxicity model.

Zebrafish are ideal models for investigating drug dose-response relationships and evaluating neurotoxicity interventions, owing to the high conservation of their GR signaling pathway with mammals (97% similarity in the DNA-binding domain and 73% in the ligand-binding domain),^22^ as well as their embryonic transparency during early development and favorable genetic tractability.^23^ Using zebrafish larvae as a model, this study found that HC induces ER calcium imbalance and mitochondrial stress by downregulating *Atp2a2*/SERCA2 function. The intervention effects of RU486 and S2A1 were verified, aiming to provide experimental evidence for elucidating the mechanism of GC-induced neurotoxicity and developing neuroprotective strategies targeting SERCA2.

## Results

### Hydrocortisone-induced toxicity and dose-response relationship in zebrafish

**Fig. 1:**
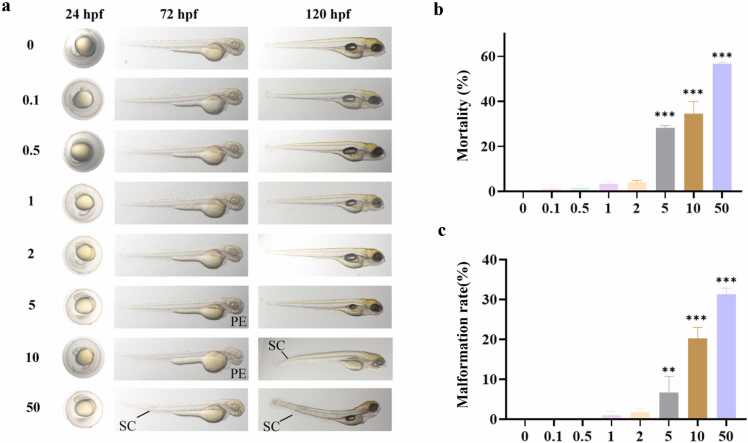
Effects of glucocorticoid (GC) exposure at different concentrations on survival rate and malformation distribution in zebrafish larvae. (a) Morphological images of zebrafish larvae after 96-hour exposure to GCs at concentrations of 0 (solvent control, 0.1% dimethyl sulfoxide), 0.1, 0.5, 1, 2, 5, 10, and 50 μM. Typical malformations: pericardial edema (PE) and spinal curvature (SC). (b) Statistical analysis of larval survival rates in each group (n = 24 larvae/group). Data are presented as the mean ± SEM from three independent experiments. (c) Distribution ratio of larval body malformations, including pericardial edema and spinal curvature, calculated based on the total number of larvae in each group. All larvae were exposed to GCs for 96 h, and statistical significance was determined by one-way ANOVA. **P < 0.01, ***P < 0.001 compared with the solvent control group.

After 96 h of exposure, GC concentration showed a positive correlation with the mortality rate of zebrafish larvae (Figure 1(b)). Mortality rates in the 0.1–2 μM groups were not significantly different from those in the 0 μM solvent control group (*P* > 0.05, one-way ANOVA), with the 2 μM group exhibiting an average mortality of 4.0% ± 0.82%. In contrast, mortality rates increased significantly in the 5, 10, and 50 μM groups (28.25% ± 0.96%, 34.5% ± 5.45%, and 56.67% ± 0.58%, respectively; *P* < 0.001). GCs induced structural malformations in zebrafish in a dose-dependent manner: exposure to concentrations ≥5 μM disrupted heart development and body axis formation, manifesting as pericardial edema and spinal curvature (Figure 1(a)). During the 96-hour exposure period, HC at concentrations of 2 μM and below did not induce acute lethal toxicity (mortality ≤ 5%) or significant structural teratogenic effects. Therefore, 2 μM HC was selected as the exposure concentration for subsequent experiments.

### Effects of hydrocortisone on the transcriptomics of zebrafish

**Fig. 2:**
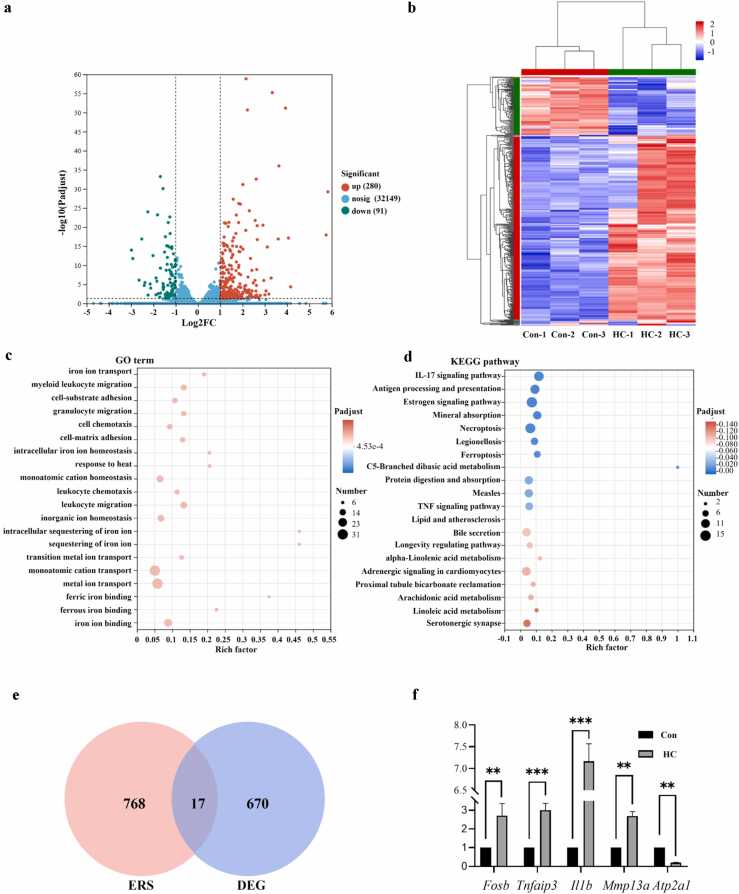
Differential gene expression profiles and functional enrichment analyses in zebrafish larvae exposed to hydrocortisone (HC). Zebrafish larvae were exposed to 2 μM HC or control (Con, 0.1% dimethyl sulfoxide) for 96 h, followed by transcriptomic analysis. (a) Volcano plot showing differential gene expression between HC-treated and Con groups. The x-axis represents the Log2FC, and the y-axis indicates the −log10 (*P*-value). (b) Heatmap depicting hierarchical clustering of differentially expressed genes (DEGs) between HC-treated and Con groups. The color gradient indicates gene expression levels, with red denoting upregulation and blue denoting downregulation. (c) Gene Ontology (GO) enrichment analysis of DEGs. The y-axis lists enriched GO terms, and the x-axis indicates the enrichment ratio (Rich Factor). Circle size reflects the number of genes associated with each GO term, while the color gradient represents statistical significance. (d) Kyoto Encyclopedia of Genes and Genomes (KEGG) pathway enrichment analysis for DEGs. The y-axis shows significantly enriched pathways, and the x-axis denotes the enrichment ratio. Circle size indicates the number of enriched genes, while the color gradient represents statistical significance. (e) Venn diagram showing overlap between ERS-related genes and DEGs. (f) Validation of gene expression levels for DEGs using qRT-PCR. Relative expression levels are presented as mean ± SEM. Statistical significance is indicated as follows: **P < 0.01, ***P < 0.001.

To investigate the effects of HC on gene expression in zebrafish, RNA-Seq was performed on larvae exposed to 2 μM HC for 96 h. Compared with the control group, a total of 371 differentially expressed genes (DEGs) were identified in the HC-treated group, including 280 upregulated and 91 downregulated genes (Figure 2(a) and (b)). Gene Ontology enrichment analysis revealed that these DEGs were significantly enriched in key biological processes such as iron metabolism, immune regulation, and cell adhesion, suggesting that GCs may influence the physiological functions of zebrafish larvae by regulating these processes (Figure 2(c)). For example, significant enrichment in iron metabolism-related processes (e.g., "iron ion transport," "iron ion binding," "intracellular iron ion homeostasis") indicates that HC may disrupt iron metabolic balance, thereby affecting cellular functions. Meanwhile, enrichment in immune-related processes (e.g., "myeloid leukocyte migration," "neutrophil migration," "leukocyte chemotaxis," "cell-matrix adhesion") suggests that GCs may alter immune homeostasis by regulating immune cell migration and adhesion. Kyoto Encyclopedia of Genes and Genomes pathway analysis further elucidated potential molecular mechanisms. DEGs were significantly enriched in the IL-17 signaling pathway, antigen processing and presentation pathway, and pathways related to ferroptosis and necroptosis. Additionally, enrichment in lipid metabolism-related pathways (e.g., arachidonic acid metabolism, linoleic acid metabolism) suggests that GCs may impair energy metabolism by disrupting lipid metabolic homeostasis (Figure 2(d)).

To further explore GC-induced molecular mechanisms, an intersection analysis of DEGs and ERS-related genes identified 17 overlapping DEGs (Figure 2(e)), among which *Atp2a2* showed the most significant expression change. To validate the transcriptome results, qRT-PCR was used to analyze the expression levels of five genes: FOSB, tnfaip3, il1b, mmp13a, and *Atp2a2* (Figure 2(f)). Compared with the control group, *Fosb*, *Tnfaip3*, and *Il1b* were significantly upregulated in the HC-treated group, while *Mmp13a* and *Atp2a2* were significantly downregulated. These results were consistent with the transcriptome trends.

### Effects of hydrocortisone on cellular calcium levels and mitochondrial function

**Fig. 3:**
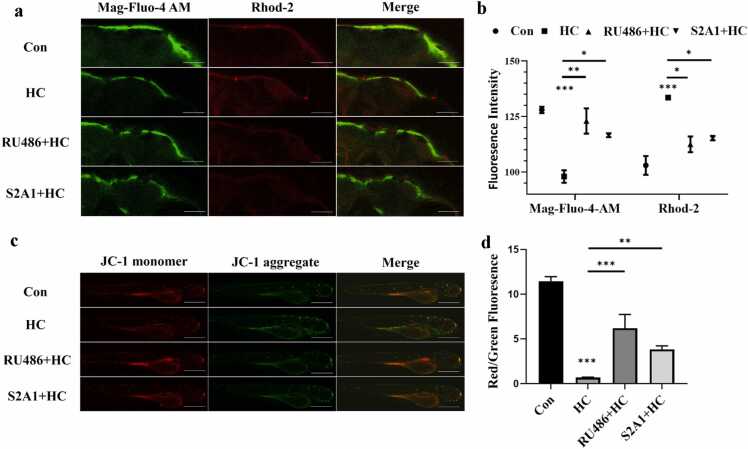
Effects of RU486 and S2A1 on calcium homeostasis and mitochondrial membrane potential in HC-treated zebrafish larvae. (a) Fluorescence staining images of zebrafish larvae (120 hpf) in different treatment groups, focusing on the brain region: green fluorescence represents cytoplasmic Ca²⁺ labeled by Mag-Fluo-4 AM, red fluorescence represents mitochondrial Ca²⁺ labeled by Rhod-2, and the merged image shows the colocalization of the two. The merged image illustrates the colocalization of the two signals. (b) Quantitative analysis of the fluorescence intensities of Mag-Fluo 4 AM and Rhod-2 (n = 3 replicates, N = 3 biological replicates, 15 embryos per replicate. Data are presented as mean ± SEM, *P < 0.05, **P < 0.01, ***P < 0.001). (c) Representative images of zebrafish larvae stained with JC-1 to assess mitochondrial membrane potential (ΔΨm). Red fluorescence is the aggregated state of JC-1 (normal membrane potential), and green fluorescence is the monomer of JC-1 (membrane potential depolarization), with merged images representing the balance of the two signals. (d) Quantification of the JC-1 red/green fluorescence ratio, reflecting changes in mitochondrial membrane potential across different groups (n = 3 replicates, N = 3 biological replicates, 15 embryos per replicate; data are presented as mean ± SEM, *P < 0.05, **P < 0.01, ***P < 0.001).

Since the ER is one of the primary intracellular calcium storage sites, the Ca²⁺ indicator Mag-Fluo-4 AM was used to monitor ER calcium levels in zebrafish larvae following HC treatment. After 96 h of exposure to 2 μM HC, ER Ca²⁺ levels decreased significantly (Figure 3(a) and (b)). Concomitantly, HC exposure led to a significant increase in mitochondrial Ca²⁺ levels, suggesting that calcium released from the ER into the cytoplasm was subsequently taken up by mitochondria in excess.

To assess the impact of HC on mitochondrial function, JC-1 dye was used to monitor mitochondrial membrane potential (MMP). JC-1 exists as aggregates (emitting red fluorescence) in mitochondria with normal membrane potential but dissociates into monomers (emitting green fluorescence) during depolarization. Following HC treatment, MMP in zebrafish larvae was significantly depolarized, and green fluorescence intensity increased (Figure 3(c) and (d)), indicating impaired mitochondrial function and potential cellular stress. These results demonstrate that HC may disrupt intracellular calcium homeostasis, leading to dysfunction of the ER and mitochondria, thereby altering normal cellular physiology.

### Hydrocortisone-mediated neuroinflammatory response and its neurotoxic effects

**Fig. 4:**
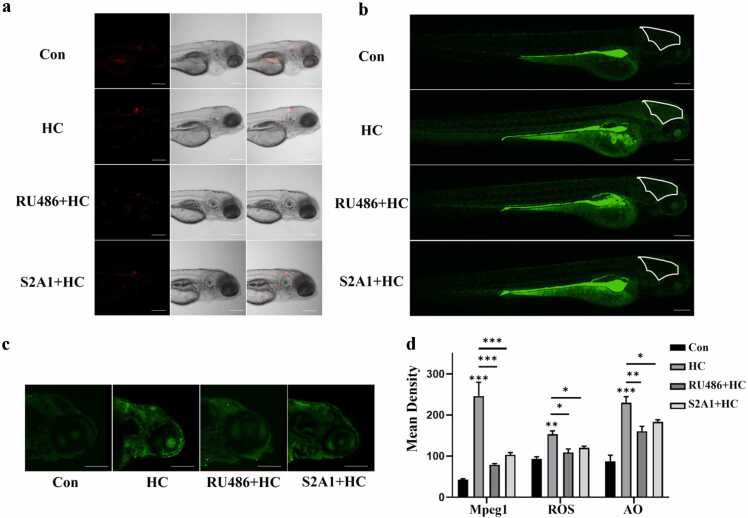
Effects of hydrocortisone treatment on macrophages, reactive oxygen species (ROS) levels, and lysosomal function in the zebrafish larval brain. (a) Representative images showing macrophages in the zebrafish brain. Macrophages are labeled with mpeg1: mCherry (red fluorescence), illustrating their distribution across different treatment groups. (b) Detection of reactive oxygen species (ROS) levels. ROS were stained using DCFH-DA (green fluorescence) to visualize their distribution in the different treatment groups. The white-boxed region indicates the brain area analyzed for fluorescence. (c) Evaluation of lysosomal function in the zebrafish brain. Acridine Orange (AO) staining (green fluorescence) was used to detect lysosomal activity, showing the fluorescence intensity and distribution in the various treatment groups. (d) Quantitative analysis of mpeg1 expression, ROS levels, and AO fluorescence intensity. Bar graphs represent the mean fluorescence density of macrophage marker expression (mpeg1), ROS levels, and lysosomal activity across different treatment groups (n = 3 replicates, N = 3 biological replicates, 15 embryos per replicate; data are presented as mean ± SEM, *P < 0.05, **P < 0.01, ***P < 0.001).

In the brains of HC-treated zebrafish larvae, there was a significant increase in macrophages, along with elevated reactive oxygen species (ROS) levels and apoptosis. Compared with the control group, expression of the macrophage marker mpeg1 in the brain was significantly upregulated (Figure 4(a)), suggesting that HC may activate macrophages to release inflammatory factors, contributing to neuroinflammation.

The positive control group (treated with 1 mM H₂O₂) showed significantly increased ROS fluorescence intensity (Figure S2), validating the sensitivity and efficacy of the detection system. Further experiments revealed that RU486 (a GC receptor antagonist) or SERCA2 activators alleviated HC-induced neuroinflammation and neurotoxicity. Specifically, mpeg1 fluorescence intensity in the brain was reduced, and ROS levels and apoptosis were significantly decreased (Figure 4(a)-(c)). These findings indicate that RU486 blocks HC-induced macrophage activation and subsequent inflammatory responses by inhibiting GC receptor overactivation, whereas SERCA2 activators may mitigate ROS accumulation and neuronal apoptosis caused by mitochondrial calcium overload by restoring calcium homeostasis, thereby ameliorating neurotoxicity.

### Effects of hydrocortisone on neurobehavioral function in zebrafish larvae

**Fig. 5:**
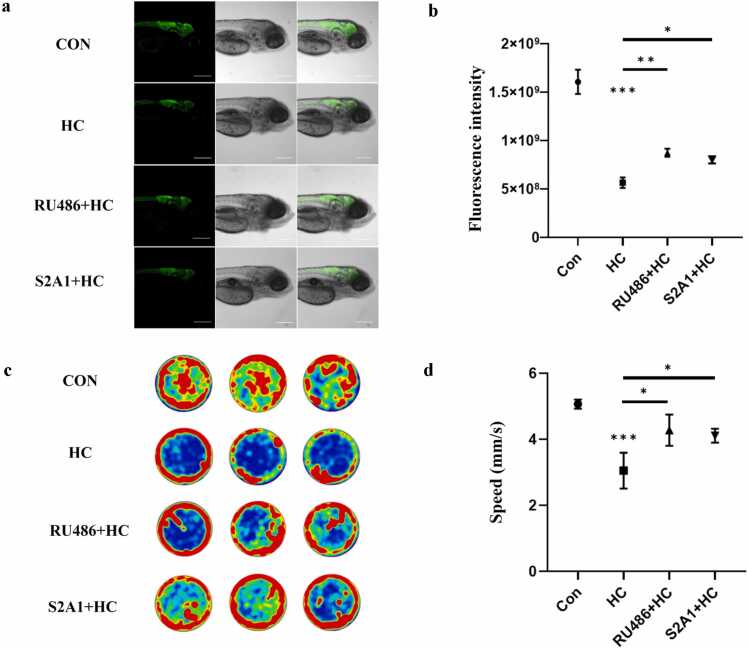
Effects of hydrocortisone on neuronal fluorescence signals and locomotor activity in zebrafish larvae. (a) Fluorescence imaging of transgenic zebrafish larvae (120 hpf). HuC-labeled neurons (green fluorescence) demonstrate the distribution and fluorescence intensity of neurons in the brain and spinal cord across different groups. (b) Quantification of neuronal fluorescence intensity. The y-axis represents fluorescence intensity. n = 3 replicates, N = 3 biological replicates, 15 embryos per replicate. Data are presented as mean ± SEM, *P < 0.05, **P < 0.01, ***P < 0.001. (c) Representative behavioral heatmap of zebrafish larvae. The movement trajectories and activity distribution of juvenile fish, with color intensity reflecting movement frequency (red indicates high-frequency activity zones, and blue indicates low-frequency activity zones). (d) Quantification of locomotor speed in zebrafish larvae (n = 3 replicates, N = 3 biological replicates, 6 embryos per replicate). The y-axis represents swimming speed (mm/s), and data are presented as mean ± SEM. Significance levels: *P < 0.05, ***P < 0.001.

To investigate the effects of HC on the nervous system, fluorescence imaging and quantitative analysis of neurons were performed using the neuron-specific marker HuC (a conserved marker of neuronal differentiation and maturation in vertebrates, whose fluorescence intensity and number of positive cells reflect neuronal survival and developmental status). Results showed that the fluorescence signal of HuC-positive neurons in the brain and spinal cord of HC-treated larvae was significantly weakened (Figure 5(a)); quantitative analysis further revealed a significant reduction in the number of HuC-positive neurons and their fluorescence intensity compared with the control group (Figure 5(b)), indicating that HC exposure may impair the structural integrity of the nervous system.

Behavioral analysis showed that HC treatment significantly reduced the locomotor ability of zebrafish larvae. At 120 hpf, the 2 μM HC group exhibited significant decreases in movement distance, duration, and average speed compared with the control group (Fig. 5C and D), suggesting marked neurological dysfunction. Additionally, RU486 and SERCA2 activators alleviated HC-induced neurobehavioral deficits to varying degrees: compared with the HC-only group, the RU486 + HC and SERCA2 activator + HC groups showed improvements in movement distance, duration, and average speed. These results indicate that both RU486 and SERCA2 activators can partially protect the nervous system from HC-induced toxic effects.

## Discussion

This study utilized a zebrafish model to investigate HC-induced neurotoxicity and its underlying molecular mechanisms. Transcriptome sequencing revealed that the *Atp2a2* gene was significantly downregulated in zebrafish following HC treatment, indicating its potential role in neurotoxicity. Functional experiments demonstrated that HC exposure disrupted calcium homeostasis, characterized by reduced ER Ca²⁺ levels and increased mitochondrial Ca²⁺ levels. This imbalance triggered MMP depolarization, elevated ROS production, and apoptosis, ultimately leading to neuronal damage and behavioral abnormalities. Intervention with the GR antagonist RU486 or SERCA2 activator partially restored calcium homeostasis, reduced ROS generation and apoptosis, and improved locomotor behavior. These findings support the hypothesis that *Atp2a2* downregulation and calcium homeostasis disruption are key drivers of HC-induced neurotoxicity, highlighting SERCA2 activator as a promising candidate for further exploration.

*Atp2a2* is a critical member of the sarco/endoplasmic reticulum calcium ATPase (SERCA) family, responsible for transporting cytoplasmic Ca²⁺ into the ER to maintain ER calcium stores and intracellular calcium homeostasis.^24^, ^25^, ^26^ In this study, significant downregulation of *Atp2a2* expression reduced ER Ca²⁺ levels, triggering an ER stress response. Previous studies have shown that calcium homeostasis disruption can activate ER stress signaling pathways (e.g., C/EBP homologous protein, GRP78) to promote apoptosis.^27^, ^28^ Excess cytoplasmic Ca²⁺ is taken up by mitochondria, causing MMP loss and excessive ROS production, which exacerbates cellular damage. This mitochondrial dysfunction impairs cellular energy production, aggravates oxidative stress, and further promotes apoptosis and neuronal injury.^29^, ^30^ Notably, mitochondrial dysfunction and oxidative stress are also linked to neuronal damage in conditions such as eating disorders,^31^ and neurodegenerative diseases (e.g., Parkinson’s disease),^32^, ^33^, ^34^ indicating that metabolic disturbances significantly impact neuronal integrity and underscoring mitochondrial dysfunction as a central driver of neural vulnerability. Additionally, in Darier disease, mutations in the *Atp2a2* gene (human homolog) cause abnormal ER calcium homeostasis^35^, ^36^; the neuropsychiatric symptoms of this disorder (e.g., cognitive impairment, epilepsy) overlap strongly with the behavioral phenotypes observed in our HC-treated zebrafish model. This suggests that calcium-ER stress axis abnormalities may represent a common pathological basis across these conditions.

Behavioral experiments revealed that HC-exposed zebrafish exhibited a significant reduction in locomotor activity, indicating impaired neuronal function. Concurrently, fluorescence imaging demonstrated a marked decrease in the number of neurons in the brain and spinal cord of HC-treated larvae. Previous studies have identified calcium homeostasis imbalance as a critical driver of neuronal dysfunction, which can disrupt synaptic transmission, alter neuronal membrane potentials, and impair firing frequencies—ultimately compromising normal nervous system function.^37^ Additionally, interactions between calcium homeostasis imbalance and mitochondrial dysfunction have been reported to further perturb neuronal excitability and induce motor deficits,^38^, ^39^, ^40^, ^41^ a relationship consistent with the GC-induced neurological impairment observed in this study. For instance, in neurodegenerative diseases such as Alzheimer’s disease, mitochondrial dysfunction is recognized as a key precursor to neuronal damage. It contributes to pathogenesis by promoting Tau protein and β-amyloid aggregation, exacerbating neurotoxicity, and further impairing neuronal function.^41^, ^42^, ^43^ The mitochondrial dysfunction observed in our study—accompanied by increased ROS production and apoptosis—aligns with these pathological mechanisms, reinforcing the potential role of mitochondrial dysfunction and its downstream effects in neurological damage.

Intervention experiments showed that the GR antagonist RU486 and SERCA2 activator effectively mitigated HC-induced neurotoxicity, suggesting protective potential against GC-related neurotoxicity. This finding provides preliminary insights for developing interventions targeting GC-associated side effects. Compared to traditional antioxidants or ER stress inhibitors, SERCA2 activator may exert protective effects by directly regulating calcium homeostasis and ER function, representing a promising direction for further investigation.

Despite these observations, this study has limitations. First, it was primarily conducted in a zebrafish model; while suitable for neurotoxicity research, specific mechanisms may differ across species, necessitating validation in mammalian models. Second, although calcium homeostasis imbalance and ER stress are linked to GC-induced neurotoxicity, the precise mechanisms underlying these associations in distinct neural regions and cell types require deeper exploration.

## Conclusions

In summary, this study offers new molecular insights into HC-induced neurotoxicity, highlighting the potential central role of *Atp2a2*-mediated calcium imbalance and ER stress. These findings enhance our understanding of GC-induced neurotoxicity and provide a theoretical basis for exploring GC-associated side effects. Future research will investigate the applicability of these mechanisms to other GC treatments and evaluate the translational potential of SERCA2 activators, aiming to illuminate the neuropathology of long-term GC exposure.

## Materials and Methods

### Zebrafish maintenance and hydrocortisone treatment

Adult zebrafish were maintained in a recirculating water system at a constant temperature of 28.5 °C under a 14/10 h light/dark cycle. They were fed newly hatched brine shrimp at scheduled daily intervals. Male and female zebrafish spawned approximately 30 min after the onset of the light period each day. Embryos were collected within 1 h post-spawning, transferred to clean petri dishes, and gently mixed to minimize inter-group variability. Zebrafish larvae were reared in E3 medium (5 mM NaCl, 0.17 mM KCl, 0.33 mM CaCl_2_, and 0.33 mM MgSO₄). They were cultured until 24 h post-fertilization (hpf), and embryos with normal development at the same stage were selected under an optical microscope for subsequent experiments.

HC (MCE, USA) was dissolved in dimethyl sulfoxide (DMSO) (Sigma, USA) to prepare a stock solution, which was then diluted to target concentrations using E3 medium. The final concentration of DMSO in each treatment group was maintained at 0.1% (v/v). To determine the toxicity threshold of GCs in zebrafish larvae, seven concentration gradients were established based on the reported effective concentration range of GCs,^44^, ^45^, ^46^ covering subphysiological to supra-pharmacological concentrations: 0.1, 0.5, 1, 2, 5, 10, and 50 μM. Zebrafish larvae treated with E3 medium containing 0.1% DMSO served as the control group. Larvae at 24 hpf were individually housed in 24-well plates, with 24 larvae per group. The experiment was performed in triplicate, resulting in a total sample size of 576 larvae. Each well received 2 mL of the test solution, and cultures were maintained at 26.0 ± 0.2 °C for 96 h. Larval morphology was observed under a microscope, and mortality (defined as cessation of heartbeat) was recorded. The maximum nonlethal concentration with a mortality rate of ≤5% was selected for subsequent experiments.

### Gene transcription analysis

The HC treatment group was continuously exposed to 2 μM HC until 120 hpf, while the control group received an equal volume of 0.1% DMSO for the same duration. After treatment, zebrafish larvae were anesthetized by immersion in 0.03% tricaine methanesulfonate (MS-222; MCE, USA). Three biological replicates were established for both groups, with 50 larvae per replicate (totaling 300 larvae across both groups). Total RNA was extracted from each group’s samples using the Trizol Total RNA Purification Kit (Simgen Biotechnology Co., Ltd., Hangzhou, China). Following RNA concentration determination with a NanoDrop instrument (NanoDrop Technologies, Wilmington, DE, USA), library construction and sequencing were performed by Majorbio Bio-Pharm Technology Co., Ltd. (Shanghai, China) using the NovaSeq X Plus platform. Raw sequencing data were quality-controlled using fastp (https://github.com/OpenGene/fastp). Clean reads were then aligned to the zebrafish reference genome (GRCz11) in orientation mode using HISAT2 software (http://ccb.jhu.edu/software/hisat2/index.shtml). DEGs were screened with DESeq2 (Version 1.24.0) using criteria of *P*-value <0.05 and |log2(fold change)| ≥ 1. Gene Ontology enrichment analysis and Kyoto Encyclopedia of Genes and Genomes pathway annotation were conducted using Blast2GO and KOBAS software, respectively.

RT-qPCR was used to quantify transcriptional levels of DEGs and validate RNA-Seq results. cDNA was synthesized using PrimeScript™ RT Master Mix (Takara, Japan), then diluted 10-fold for use as qPCR templates. qPCR reactions were performed with AceQ qPCR SYBR Green Master Mix (Vazyme, China) following the manufacturer’s instructions. β-actin served as the internal reference gene, and relative mRNA expression levels were calculated using the 2^−ΔΔCT^ method. Primers were designed with Primer Premier 5.0 software, and sequences are provided in Table S1.

### Measurement of endoplasmic reticulum and mitochondrial Ca²⁺ levels

To detect Ca²⁺ levels in the ER and mitochondria of zebrafish following HC treatment, the experiment was designed with six groups: a control group (0.1% DMSO), an HC-treated group (2 μM), a RU486-only group (1 μM), a S2A1-only group (5 μM), a RU486 + HC cotreated group, and a S2A1 + HC cotreated group. Each group included three biological replicates, with 15 zebrafish larvae per replicate, and all larvae were exposed until 120 hpf.

After treatment, zebrafish larvae were incubated with 4 μM Mag-Fluo-4 AM (Mao Kang Biotechnology Co., Ltd.) and 2 μM Rhod-2 AM (Yeasen Biotechnology Co., Ltd.) in the dark at 28 °C for 30 min. The larvae were then washed three times in E3 medium, with each wash lasting 5 min. Observations were performed using an Olympus FV3000 confocal laser scanning microscope (Olympus, Japan). Mag-Fluo-4 AM was excited with a 488 nm laser, and fluorescence was detected at 510–530 nm. Rhod-2 AM was excited with a 561 nm laser, and emitted fluorescence was recorded at 595 nm. Fluorescence micrographs were processed using ImageJ software (NIH, USA) to quantify fluorescence intensity.

### Mitochondrial membrane potential detection

To assess the impact of HC on mitochondrial function in zebrafish, changes in MMP were detected using the JC-1 fluorescent probe (HY-15534, MCE, China). Experimental groups were identical to those described in the previous section for Ca²⁺ Levels, with larvae from each group exposed until 120 hpf. Larvae were transferred to a 96-well plate, and each well was filled with 100 μL of E3 medium containing 5 μM JC-1. They were then incubated at 28 °C in the dark for 30 min. After staining, larvae were washed three times in E3 medium before image acquisition. Fluorescence images were captured using a confocal laser scanning microscope: red fluorescence (excitation wavelength 561 nm) indicated normal MMP (JC-1 aggregate form), while green fluorescence (excitation wavelength 488 nm) indicated dissipated MMP (JC-1 monomer form). Results were expressed as the ratio of red to green fluorescence intensity and statistically analyzed using GraphPad Prism 8.

### Detection of neuroinflammation and cellular damage

To further investigate the effects of HC on macrophages in the zebrafish head, Tg(mpeg1:mCherry) transgenic larvae exposed until 120 hpf were used. The grouping criteria and processing methods were consistent with those used in the aforementioned experiments. After anesthesia with 0.03% MS-222, fluorescent images of mCherry-labeled macrophages (excitation/emission: 561/617 nm) were acquired *via* confocal microscopy. The number of macrophages was quantified using ImageJ software.

For ROS detection, 15 larvae from each treatment group were collected after exposure to HC until 120 hpf. To validate the ROS detection system, a positive control group was included: 15 normally developing 120 hpf larvae were treated with 1 mM H₂O₂ in E3 medium for 4 h. All larvae were then incubated in 40 μM DCFH-DA (MCE, USA) working solution at 28 °C in the dark for 1 h. After three washes in E3 medium, larvae were anesthetized with 0.03% MS-222 and imaged using a confocal microscope. ROS fluorescence intensity was analyzed using Image-Pro Plus software.

Apoptotic cells were detected using acridine orange hydrochloride (AO, MCE, USA). Fifteen larvae per group were incubated in 20 μM AO working solution in the dark for 30 min. After staining, larvae were washed three times in E3 medium (5 min per wash), and apoptotic cells (exhibiting green fluorescence) were observed under a microscope.

### Neurological behavioral assessment

In Tg (HuC:EGFP) transgenic zebrafish, EGFP is specifically expressed in neurons under the HuC promoter, serving as a direct fluorescent marker for neurons. Changes in neural development were evaluated by monitoring green fluorescent signals in the central nervous system of Tg(HuC:EGFP) transgenic zebrafish. At 120 hpf, larvae from different treatment groups were anesthetized, and fluorescent images were captured *via* confocal microscopy. Green fluorescent protein intensity was quantified using ImageJ software (n = 15 per group).

The effects of HC on neural function were assessed using FishTrack software (XinRuan Technology, China). At 168 hpf, three larvae per group were placed individually in a 24-well plate (1 mL E3 medium per well) and adapted to the behavior analysis system for 5 min. Behavioral tracking was then conducted for 30 min. Total movement distance, average speed, and cumulative movement time were extracted and analyzed.

### Statistical analysis

One-way ANOVA and Tukey’s multiple comparisons test were used to analyze the results. The data were presented as mean ± SEM. Statistical differences among groups were considered to be significant when P value was less than 0.05 or 0.01.

## CRediT authorship contribution statement

**Lei Feng:** Writing – review & editing. **Shunxin Hu:** Investigation. **Ben Sang:** Validation. **Xinglu Miao:** Validation. **Pei Jiang:** Data curation, Conceptualization. **Weihua Kong:** Writing – original draft, Investigation.

## Author contributions

Weihua Kong performed the experiments, analyzed the data, and drafted the manuscript. Pei Jiang, Xinglu Miao, Ben Sang, and Shunxin Hu helped with experimental design and discussion. Lei Feng developed the concept and provided guidance during the study. All authors contributed to the manuscript in conceptualization and approved the final manuscript.

## Declarations of interest

The authors declare that they have no known competing financial interests or personal relationships that could have appeared to influence the work reported in this paper.

## Data Availability

Data will be made available on request.

## Footnotes

Appendix A Supplementary data associated with this article can be found online at doi:10.1016/j.cstres.2025.100112.
