# Supplementary material for: The role of Atp2a2-mediated calcium imbalance and endoplasmic reticulum stress in hydrocortisone-induced neurotoxicity
Source: Cell Stress Chaperones. 2025 Sep 17;30(6):100112. doi: 10.1016/j.cstres.2025.100112 (PMC12509107; doi:10.1016/j.cstres.2025.100112)
Supplement: Supplementary file 1 — Supplementary material [file mmc1.docx]

**Table S1.** The Sequences of primer list used in this study

| Gene name | Forward Primer | Reverse Primer |
| --- | --- | --- |
| *Atp2a2* | TGACCTTCGTTGGCTGTGTTGG | AGCCTTGCCAGTCACATCCTCA |
| *Fosb* | TCTCAGTACCTGTCGTCCGT | CCAGTGGCTGTTACACACTC |
| *Tfaip3* | ATGATCCGAATGAGCCAGTT | AATTCCCATCTCCGTTGGTT |
| *I1b* | TCCAAACGGATACGACCAGC | GCGGTGCTGATAAACCAACC |
| *Mp13a* | AAGGTTTGGGCTCTCTATGG | GGAAAACCTTTATCCATTTGCC |
| *β-actin* | ACGAACGACCAACCTAAACTCT | TTAGACAACTACCTCCCTTTGC |

**
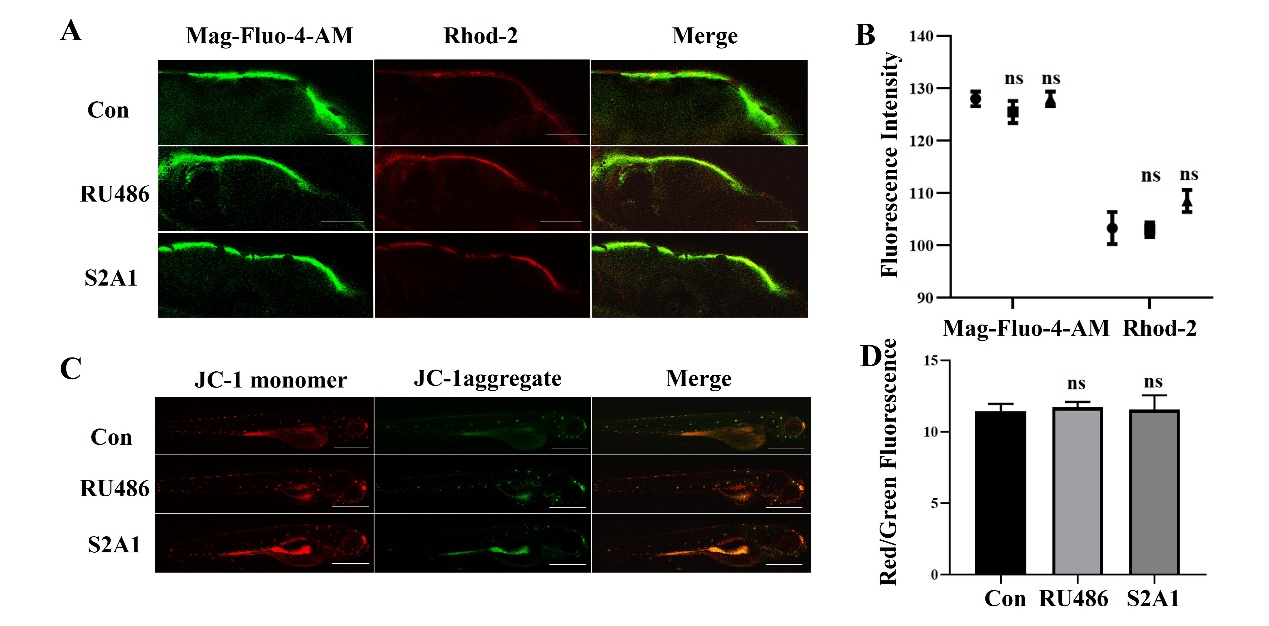
**

**Figure S1.** Effects of RU486 and S2A1 on calcium homeostasis and mitochondrial membrane potential in zebrafish larvae. (A) Fluorescence staining images of zebrafish larvae (120 hpf) in different treatment groups, focusing on the brain region: green fluorescence represents cytoplasmic Ca²⁺ labeled by Mag-Fluo-4 AM, red fluorescence represents mitochondrial Ca²⁺ labeled by Rhod-2, and the merged image shows the co-localization of the two. The merged image illustrates the co-localization of the two signals. (B) Quantitative analysis of the fluorescence intensities of Mag-Fluo 4 AM and Rhod-2 (n = 3 replicates, N = 3 biological replicates, 15 embryos per replicate. Data are presented as mean ± SEM, *P < 0.05, **P < 0.01, ***P < 0.001). (C) Representative images of zebrafish larvae stained with JC-1 to assess mitochondrial membrane potential (ΔΨm). Red fluorescence is the aggregated state of JC-1 (normal membrane potential), green fluorescence is the monomer of JC-1 (membrane potential depolarization), with merged images representing the balance of the two signals. (D) Quantification of the JC-1 red/green fluorescence ratio, reflecting changes in mitochondrial membrane potential across different groups. (n = 3 replicates, N = 3 biological replicates, 15 embryos per replicate. Data are presented as mean ± SEM, ns not statistically significant).

**
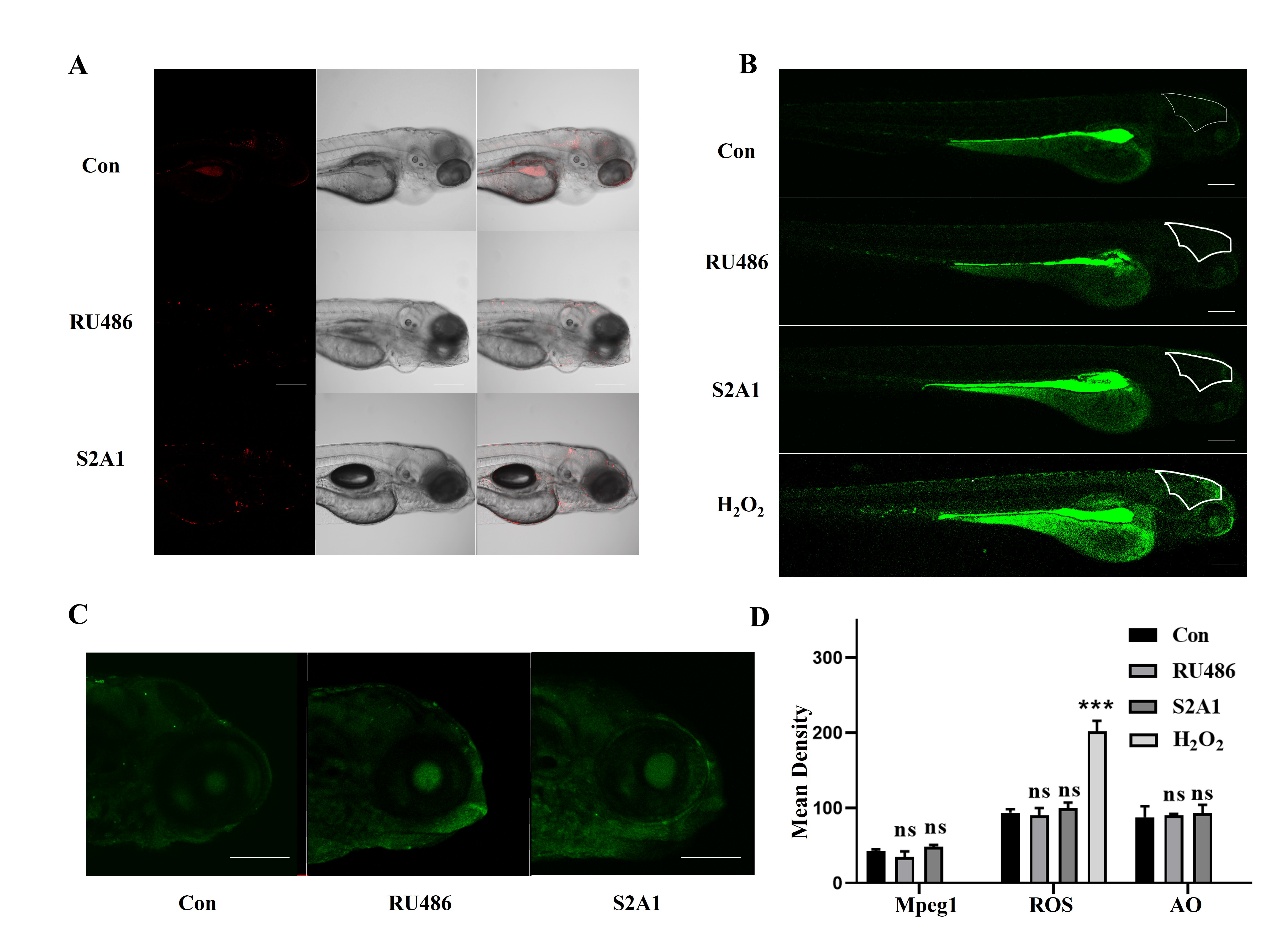
**

**Figure S2.** Effects of RU486 and S2A1 treatment on macrophages, reactive oxygen species (ROS) levels, and lysosomal function in the zebrafish larval brain. (A) Representative images showing macrophages in the zebrafish brain. Macrophages are labeled with mpeg1: mCherry (red fluorescence), illustrating their distribution across different treatment groups. (B) Detection of reactive oxygen species (ROS) levels. ROS were stained using DCFH-DA (green fluorescence) to visualize their distribution in the different treatment groups. The white-boxed region indicates the brain area analyzed for fluorescence. (C) Evaluation of lysosomal function in the zebrafish brain. Acridine Orange (AO) staining (green fluorescence) was used to detect lysosomal activity, showing the fluorescence intensity and distribution in the various treatment groups. (D) Quantitative analysis of mpeg1 expression, ROS levels, and AO fluorescence intensity. Bar graphs represent the mean fluorescence density of macrophage marker expression (mpeg1), ROS levels, and lysosomal activity across different treatment groups. (n = 3 replicates, N = 3 biological replicates, 15 embryos per replicate. Data are presented as mean ± SEM, ***P < 0.001, ns not statistically significant).

**
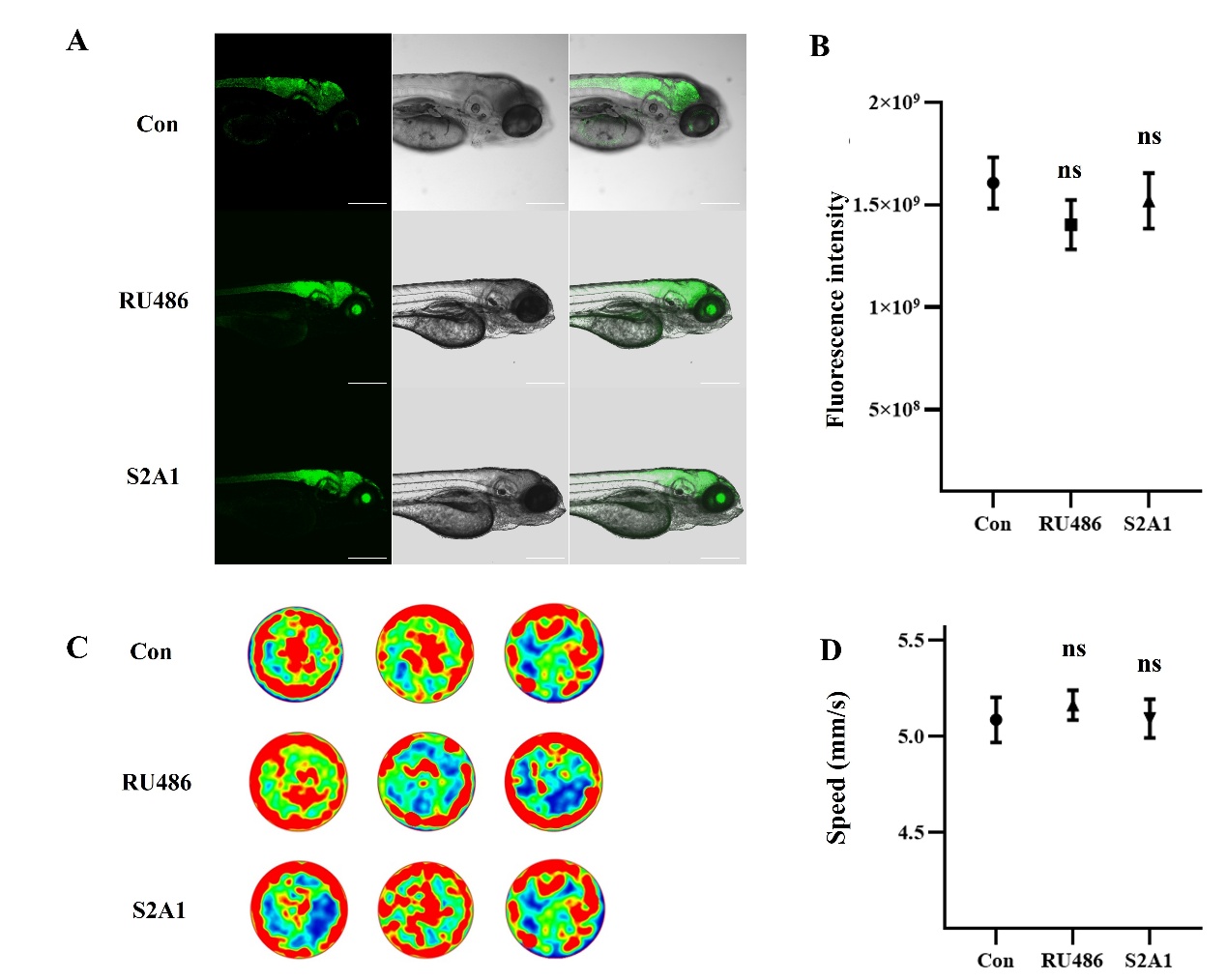
**

**Figure S3.** Effects of RU486 and S2A1 on neuronal fluorescence signals and locomotor activity in zebrafish larvae. (A) Fluorescence imaging of transgenic zebrafish larvae (120 hpf). HuC-labeled neurons (green fluorescence) demonstrate the distribution and fluorescence intensity of neurons in the brain and spinal cord across different groups. (B) Quantification of neuronal fluorescence intensity. The y-axis represents fluorescence intensity. n = 3 replicates, N = 3 biological replicates, 15 embryos per replicate. Data are presented as mean ± SEM, *P < 0.05, **P < 0.01, ***P < 0.001. (C) Representative behavioral heatmap of zebrafish larvae. The movement trajectories and activity distribution of juvenile fish, with color intensity reflecting movement frequency (red indicates high-frequency activity zones, blue indicates low-frequency activity zones). (D) Quantification of locomotor speed in zebrafish larvae (n = 3 replicates, N = 3 biological replicates, 6 embryos per replicate). The y-axis represents swimming speed (mm/s), and data are presented as mean ± SEM. Significance levels: ns not statistically significant.
